# Efficient AAV9 Purification Using a Single-Step AAV9 Magnetic Affinity Beads Isolation

**DOI:** 10.3390/ijms25158342

**Published:** 2024-07-30

**Authors:** Kian Chuan Sia, Zhen Ying Fu, Siti Humairah Mohd Rodhi, Joan Hua Yi Yee, Kun Qu, Shu Uin Gan

**Affiliations:** 1 Phoenix Laboratory of Gene Therapy and Cell Therapy, Department of Surgery, Yong Loo Lin School of Medicine, National University of Singapore, MD11, Basement 1, 10, Medical Drive, Singapore 117597, Singapore; sursiak@nus.edu.sg (K.C.S.); surfz@nus.edu.sg (Z.Y.F.); surshmr@nus.edu.sg (S.H.M.R.); 2 Infectious Diseases Translational Research Programme, Department of Biochemistry, Yong Loo Lin School of Medicine, National University of Singapore, Singapore 117545, Singapore; e0559166@u.nus.edu (J.H.Y.Y.); kqu@nus.edu.sg (K.Q.)

**Keywords:** adeno-associated virus serotype 9, AAV9 magnetic affinity beads, single-step AAV9 purification, cost-effective, no endonuclease treatment, small-scale purification, reusable magnetic beads, scalable, in vivo bioactivity, efficient

## Abstract

Adeno-associated viruses (AAVs) have emerged as promising tools for gene therapy due to their safety and efficacy in delivering therapeutic genes or gene editing sequences to various tissues and organs. AAV serotype 9 (AAV9), among AAV serotypes, stands out for its ability to efficiently target multiple tissues, thus holding significant potential for clinical applications. However, existing methods for purifying AAVs are cumbersome, expensive, and often yield inconsistent results. In this study, we explore a novel purification strategy utilizing Dynabeads™ CaptureSelect™ magnetic beads. The AAV9 magnetic beads capture AAV9 with high specificity and recovery between 70 and 90%, whereas the AAVX magnetic beads did not bind to the AAV9. Through continuous interaction with AAVs in solution, these beads offer enhanced clearance of genomic DNA and plasmids even in the absence of endonuclease. The beads could be regenerated at least eight times, and the used beads could be stored for up to six months and reused without a significant reduction in recovery. The potency of the AAV9-purified vectors in vivo was comparable to that of iodixanol purified vectors.

## 1. Introduction

Adeno-associated viruses (AAVs) are small, single-stranded, non-enveloped viruses with an icosahedral capsid spanning approximately 25 nm in diameter. They have emerged as promising vectors for gene therapy due to their safety profile and effectiveness in delivering stable expression of therapeutic replacement genes or gene editing sequences to different tissues and organs [1].

Among AAV serotypes, AAV serotype 9 (AAV9) has garnered significant attention for its ability to efficiently target various tissues such as Central Nervous System (CNS) [2], smooth muscle [3], skeletal muscle [4], lung [5], liver [6], cardiovascular [7], pancreas [8], retina [9], inner ear [10], testes [11], kidney [12], and adipose tissue [13], making it a valuable candidate for clinical translations. To date, there is one Food and Drug Administration (FDA)-approved AAV9 therapeutic, Onasemnogene Abeparvovec, for spinal muscular atrophy (SMA) treatment, and over 20 clinical trials involving the use of AAV9 in treating human Batten’s disease, Canavan disease, Duchenne Muscular Dystrophy, Pompe Disease, NGLY1 Deficiency, and muscle and heart diseases (see Table S1 with AAV9 clinical trials information).

The different purification methods of AAVs to date have been laborious, costly, and often yield inconsistent results. Current research-grade methods involve numerous steps, including caesium chloride (CsCl) or iodixanol density gradient ultracentrifugation, which are effective in the separation of the full genome and empty particles but have several drawbacks. These include the requirement for specialized equipment and reagents, making them impractical for large-scale production [14]. Variability in yields and purity is often observed, making it challenging to ensure a consistent product.

To address these limitations, researchers have explored alternative purification methods such as Polyethylene glycol (PEG) precipitation followed by chloroform extraction that are less laborious and cost-effective [15,16]. These are good for rapid screening purposes without requiring AAVs of high purity. The recent introduction of AAV affinity resin-packed columns in chromatography, such as Sepharose-AVB HP, Capto-AVB (Cytiva, Uppsala, Sweden), AVRI-AAV2, 5, 8, or 9 (Repligen, Waltham, MA, USA), and the POROS CaptureSelect™ AAV8, AAV9, and AAVX (Thermo Fisher Scientific, Waltham, MA, USA), represents a significant step forward in improving the purity and yield of AAVs. AVB resins have been shown to bind to serotypes 1, 2, 3, and 5 [17] and AAVX affinity resin, which was known to efficiently capture a panel of 15 divergent AAV serotypes, including the commonly used AAV2, AAV8, AAV9, PHP.B, and Anc80 [18].

This study explores the use of a new form of AAV affinity beads known as Dynabeads™ CaptureSelect™ magnetic beads. The Dynabeads™ are non-porous monodisperse 4.5 μm superparamagnetic beads. The AAVX and AAV9 ligands that are coupled to the beads are identical to those used in the POROS CaptureSelect™. They are highly mobile in solution, enabling ligands coupled to the beads to continuously interact with the virus in the tube. DynaBeads CaptureSelect AAVX magnetic Beads are magnetic affinity particles for the purification of a broad range of naturally occurring and synthetic AAV serotypes, whereas the AAV9 beads are specific to AAV9. The capture and washes in free solution allow for better clearance of genomic DNA (gDNA), plasmids, and host cell proteins. We hypothesized that endonuclease treatment may not be crucial or necessary for purification.

There are no detailed reports on the use of the Dynabeads for AAV purification to date; hence, we evaluated and optimized the capture of AAV9 using AAVX and AAV9 Dynabeads on a small-scale initially and subsequently scaled up the AAV9 beads purification by 40 times. We optimized and tested the purification process in the presence or absence of the endonuclease treatment. Binding capacities and varying parameters such as incubation time and sample volume for the AAV9-CAG-GFP purified virus and viral supernatant were measured at the recommended scale of 40 μL of beads slurry. We scaled up the capture by 40 times using crude supernatant harvest and assessed the residual genomic host cell DNA levels. The preparations were also compared to the gold standard prepared by iodixanol. The results are presented in this manuscript.

## 2. Results

### 2.1. Effective AAV9 Purification from Crude Viral Supernatant and DNA Contamination Removal with Magnetic Affinity Beads, Eliminating the Need for Endonuclease Treatment

In our initial efforts to purify AAV9-CAG-GFP from endonuclease-treated viral supernatant, we employed a POROS AAVX affinity column (Thermo Fisher Scientific). However, this approach yielded only a modest recovery rate of approximately 35.4% AAV in the elution phase, accompanied by notable losses: 17.6% AAV across three wash steps and an additional 17.4% in the flowthrough (refer to Figure S1 for details). In response, we sought a more efficient purification method and turned to AAVX and AAV9 magnetic beads for their claims of simplicity and speed.

Our initial attempts with AAVX magnetic beads were unsuccessful in capturing AAV9 particles (refer to Figure S2). Consequently, we redirected our focus towards utilizing AAV9 magnetic beads for the purification process.

**Figure 1:**
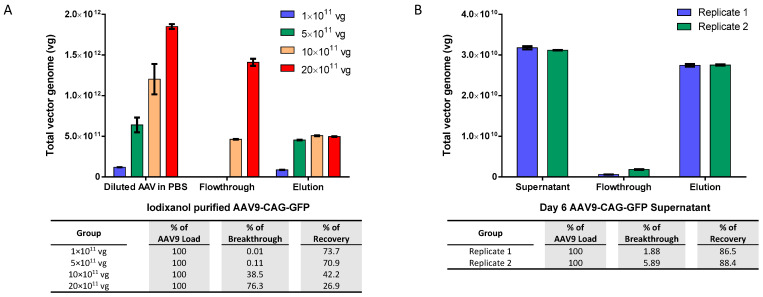
Adeno-associated virus serotype 9 (AAV9) magnetic affinity beads evaluation. (**A**) Re-purification of iodixanol-purified AAV9-CAG-GFP, diluted to virus concentrations ranging from 1 × 10^11^ to 20 × 10^11^ total vector genomes (vg) in 500 µL phosphate buffered saline (PBS). (**B**) Purification of AAV9-CAG-GFP viral supernatant, containing approximately 3 × 10^10^ total vg of AAV9 in serum-free Dulbecco’s Modified Eagle Medium (SF-DMEM). Each purification used 0.5 mL of AAV9 viral solution and 40 µL of magnetic beads. Breakthrough and recovery percentages are indicated in the table below. Data are presented as mean (SD) (*n* = 3).

In order to determine the maximum binding capacity of the AAV9 magnetic affinity beads without confounding factors such as inhibition of impurities in culture medium, we initially evaluated the AAV9 beads using an increasing amount of iodixanol-purified AAV9-CAG-GFP in 0.5 mL of phosphate buffered saline (PBS) as the sample (Figure 1A). The results showed that the maximum binding capacity for 40 µL (equivalent to 1.2 mg) of slurry was approximately 5 × 10^11^ vector genomes (vg) of AAV9, which is equivalent to about 4.2 × 10^11^ vg per milligram of beads. We observed almost no breakthrough in the flowthrough when the total AAV9 load was 5 × 10^11^ vg or less, indicating the efficient binding of AAV9 by the magnetic affinity beads. Breakthrough was only observed in the flowthrough when the total AAV load exceeded the binding capacity of the beads. After elution with 50 mM citric acid at pH 2.5, more than 70% of the AAV9 was successfully recovered when the total AAV9 load was 5 × 10^11^ vg or less.

Next, we evaluated the potential of the beads to purify AAV9 crude viral supernatant. Similar to the observations when purifying iodixanol-purified AAV9, the magnetic beads were also found to purify about 3.15 × 10^10^ vg of AAV9 crude viral supernatant with very low breakthrough in the flowthrough and more than 85% recovery (approximately 2.75 × 10^10^ vg) in elution, as shown in Figure 1B.

**Figure 2:**
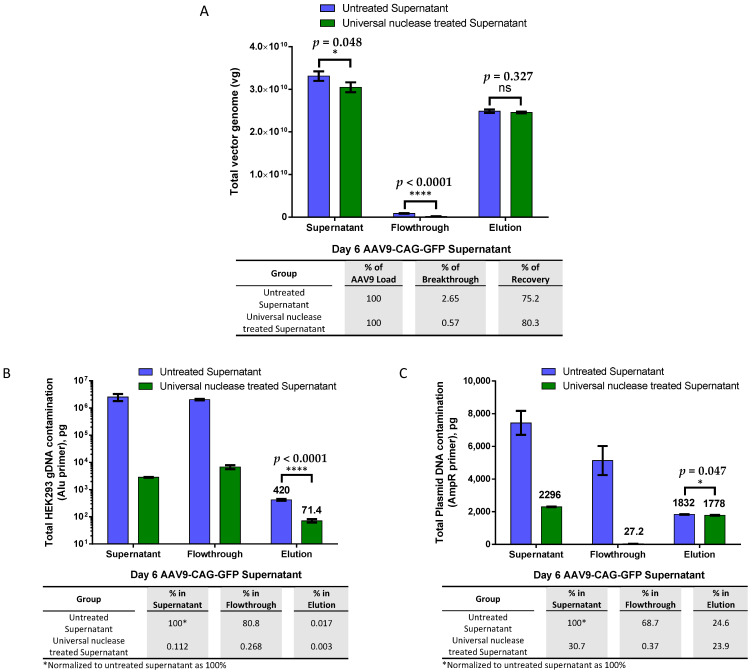
AAV9 magnetic affinity beads purification with and without endonuclease treatment. (**A**) Comparison of magnetic affinity beads purification of AAV9-CAG-GFP viral supernatant with and without endonuclease treatment. Breakthrough and recovery percentages are indicated in the table below. (**B**) Total human embryonic kidney 293 (HEK293) genomic DNA (gDNA) contamination. (**C**) Total plasmid DNA contamination. DNA contamination percentages (normalized to untreated supernatant as 100%) in endonuclease-treated supernatant, flowthrough, and elution are indicated in the table below. Endonuclease treatments: 90 U/mL endonuclease with 2 mM magnesium chloride (MgCl_2_) for 30 min in a 37 °C water bath. Data are presented as mean (SD) (*n* = 3). Statistical significance was determined using Student’s unpaired *t*-test, where ‘ns’ indicates not significant, * indicates *p* < 0.05 and is considered statistically significant, and **** indicates *p* < 0.0001 and is considered extremely significant.

We then compared the effectiveness of AAV9 magnetic affinity beads in purifying untreated versus endonuclease-treated viral crude supernatant to understand whether host gDNA and plasmid DNA contamination from transfection during packaging interfered with AAV9 binding to the beads. In Figure 2A, the results showed that the total vg in the viral supernatant were reduced by almost 8% after endonuclease treatment. However, this decrease did not significantly affect the total viral yield or recovery during elution. The recoveries of the total vg of the eluted AAV9 between the untreated and endonuclease-treated supernatants were not significantly different. In both groups, less than 3% of the total vg was found in the flowthrough, while more than 75% of AAV9 was recovered during elution.

The quantification of total host gDNA contamination (Figure 2B) revealed that over 99.98% of gDNA contamination was successfully removed during purification with AAV9 magnetic affinity beads when untreated crude viral supernatant was used as the starting material. When endonuclease-treated crude viral supernatant was used, gDNA contamination in the elution was further reduced by almost sixfold.

The total plasmid DNA contamination (Figure 2C) quantitative PCR (qPCR) quantification showed a very similar level of total plasmid DNA contamination (less than a 1% difference) in both elutions, although the difference was statistically significant. Endonuclease treatment degraded almost 70% of the contaminated plasmid DNA in the crude viral supernatant. Interestingly, only less than 0.5% of the remaining 30% of contaminated plasmid DNA after endonuclease treatment was detected in the flowthrough, and the remaining 30% was found to co-elute with the AAV9 particles in the final elution step.

Since endonuclease treatment showed no significant advantage but increased the cost of purification when purifying AAV9 using the magnetic affinity beads method, subsequent optimization steps were performed with crude AAV9 viral supernatant without endonuclease treatment.

### 2.2. Optimal AAV9 Magnetic Affinity Beads Utilization for Maximum AAV9 Purification Yield with Minimal Loss in Flowthrough

**Figure 3:**
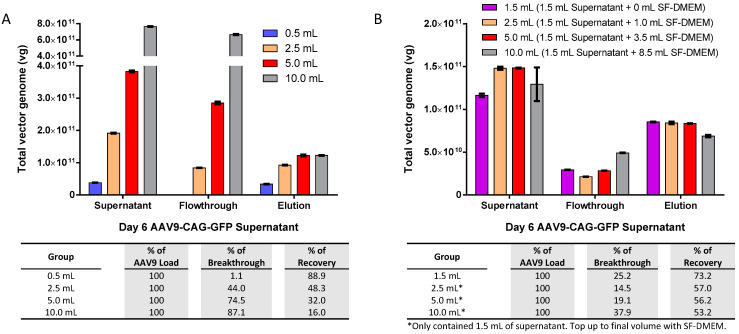

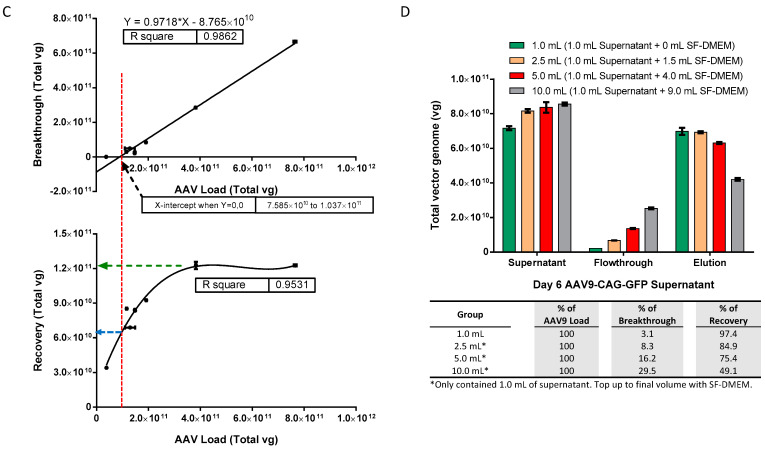
Optimizing crude viral supernatant volume and AAV load for magnetic affinity beads purification. (**A**) Effect of increasing the volume of AAV9-CAG-GFP viral supernatant from 0.5 to 10.0 mL (containing total AAV9 ranging from about 4 × 10^10^ to about 8 × 10^11^ total vg). (**B**) Effect of increasing the volume of viral supernatant from 1.5 to 10.0 mL containing a 1.5 mL fixed volume of AAV9-CAG-GFP (containing about 1.2 × 10^11^ total vg of AAV9) that was topped up to the final volume with SF-DMEM. (**C**) Correlation of AAV load versus breakthrough (top panel) and recovery (bottom panel). A red dashed line marks the AAV load where breakthrough is zero, and the AAV load range is estimated using Prism 6 software (indicated by a black arrow). The blue arrow indicates the corresponding AAV9 recovery when breakthrough is zero, while the green arrow indicates the maximum capacity of AAV9 magnetic affinity beads for crude viral supernatant. (**D**) Effect of increasing the volume of viral supernatant from 1.0 to 10.0 mL containing a 1.0 mL fixed volume of AAV9-CAG-GFP (containing about 8 × 10^10^ total vg of AAV9) that was topped up to the final volume with SF-DMEM. Breakthrough and recovery percentages are indicated in the table below. Data are presented as mean (SD) (*n* = 3).

Although we have ascertained that the AAV9 beads binding capacity of iodixanol purified virus is up to 5 × 10^11^ vg in 0.5 mL PBS per 40 µL slurry, our AAV concentration from supernatant harvest is often in the range of 3–5 × 10^10^ vg/mL, which is much lower than the maximum capacity. To maximize the potential of AAV9 magnetic affinity beads for the direct purification of crude AAV9 viral supernatant, we investigated the optimal AAV9 load and volume for the magnetic affinity beads. We increased the volume of crude viral supernatant from 0.5 mL to 2.5, 5.0, and 10 mL, which corresponded to AAV9 loads of about 4 × 10^10^, 2 × 10^11^, 4 × 10^11^, and 8 × 10^11^ vg per 40 µL of slurry, respectively (Figure 3A).

The breakthrough of unbound AAV9-CAG-GFP in the flowthrough was observed in all the tested groups with AAV9 loads above 4 × 10^10^ vg, and the maximum amount of eluted AAV9 was capped at approximately 1.2 × 10^11^ vg regardless of the input amount. We performed a follow-up experiment using a fixed AAV9-CAG-GFP load of approximately 1.2 × 10^11^ vg (equivalent to 1.5 mL of crude viral supernatant) and topped up to 2.5, 5.0, and 10 mL using serum-free Dulbecco’s Modified Eagle Medium (SF-DMEM), as shown in Figure 3B. The maximum eluted AAV9 was further reduced to about 8 × 10^10^ vg or less, and an AAV9-CAG-GFP breakthrough was observed in the flowthrough of all groups.

To further interpret the results, the breakthrough and recovery data from Figure 3A,B were plotted against their corresponding AAV load in total vg and analyzed using Prism 6 software version 6.01 (GraphPad Software, Boston, MA, USA), as shown in Figure 3C. The software analysis revealed a strong linear relationship between breakthrough and AAV load (upper panel) with an R^2^ value of 0.9862. The estimated AAV load for “no breakthrough” (i.e., the X-intercept when Y = 0,0) was about 7.585 × 10^10^ to 1.037 × 10^11^ vg, with a corresponding recovery of about 6 to 7 × 10^10^ total vg. Recovery and AAV load also showed a good fit with a third-order polynomial equation (lower panel) with an R^2^ value of 0.9531. The highest recovery of about 1.2 × 10^11^ vg was achievable when the AAV load was more than 4 × 10^11^ vg. However, at this point, the AAV load exceeded the bead’s optimum capacity by approximately 3.33-fold, leading to a significant amount of breakthrough or unbound AAV9 in the flowthrough.

To investigate whether the volume of AAV9 load had any impact on magnetic affinity beads purification, we repeated the experiment using a reduced fixed load of AAV9-CAG-GFP at approximately 8 × 10^10^ vg (within the “no breakthrough” range according to the software analysis in Figure 3C). This load was equivalent to 1.0 mL of crude viral supernatant, and we topped it up to 2.5, 5.0, and 10 mL using SF-DMEM. The results indicated that the increase in the volume/beads ratio of AAV9 load interfered with the binding of AAV9 to the magnetic affinity beads and showed an increasing loss of the AAV9 in the flowthrough as the AAV load volume increased (Figure 3D). Additionally, the recovery of AAV9 in the elution showed a decreasing trend as the AAV load volume/beads ratio increased. Therefore, it is not beneficial to increase the volume beyond 0.5 mL of crude viral supernatant, and the optimal ratio is approximately 0.5 mL of crude viral supernatant for every 40 µL or 1.2 mg of AAV9 magnetic affinity beads.

### 2.3. Reusability of AAV9 Magnetic Affinity Beads

To evaluate reusability, we performed 8 rounds of repeated use of AAV9 magnetic affinity beads. We compared two cleaning methods: (1) a gentler 1-min washing step using washing buffer (PBS with 150 mM sodium chloride (NaCl)) and (2) a harsher and more thorough regenerative wash using 6M Guanidine hydrochloride (HCl) for 10 min, followed by two 1-min washes with wash buffer before the next round of purification. We determined if (1) the gentler method was sufficient for cleaning the beads for repeated use, and (2) if the harsher and more thorough regenerative wash was tolerable for the magnetic affinity beads during repeated use.

**Figure 4:**
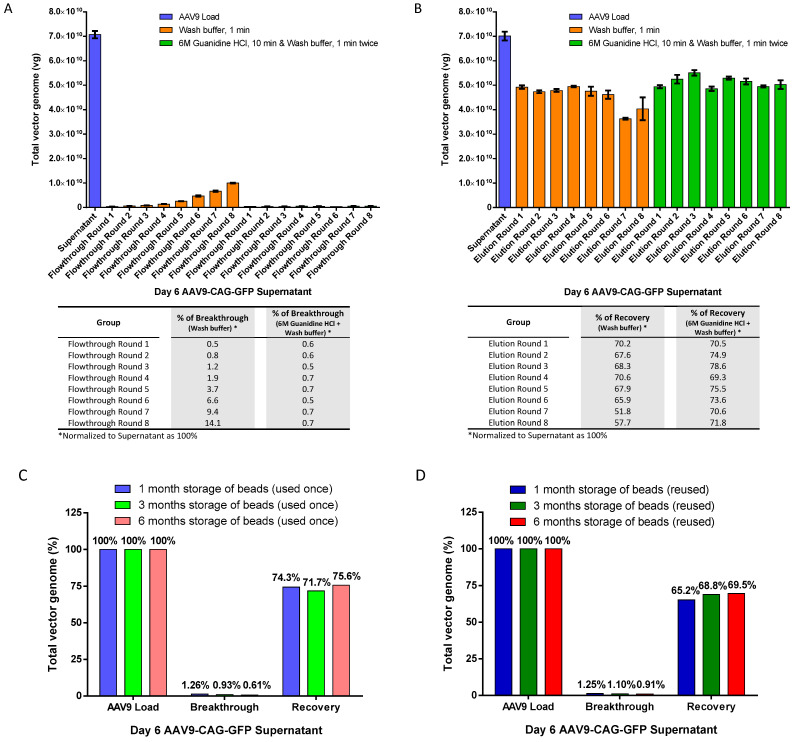
Reusability of magnetic affinity beads for AAV9 purification. (**A**) AAV9 breakthrough in flowthrough after 8 rounds of reuse. (**B**) AAV9 recovery in elution after 8 rounds of reuse. Between different rounds of purification, the beads were subjected to either 1 min of washing with wash buffer or 10 min of washing with 6M Guanidine hydrochloride (HCl), followed by two 1-min washes with wash buffer. Breakthrough or recovery percentages (normalized to supernatant as 100%) for flowthrough or elution, respectively, are indicated in the table below. (**C**) Effect of magnetic beads storage at 4 °C for 1, 3, and 6 months with AAV9 magnetic affinity beads that were used once and regenerated. (**D**) Reuse the same magnetic beads that were regenerated at each round. The beads were regenerated with 10 min of washing with 6M Guanidine HCl, followed by two 1-min washes with wash buffer, before storage in a 4 °C fridge. Breakthrough and recovery percentages are presented (normalized to AAV9 load as 100%). Data are presented as mean (SD) (*n* = 3).

The results in Figure 4A showed that a 1-min wash with washing buffer was not sufficient to clean the beads for repeated use, as the percentage of breakthrough continued to increase from 0.5% to 14.1% by round 8. On the other hand, the use of 6M Guanidine HCl followed by washing twice with wash buffer proved to be more effective in cleaning the beads and prepared them for subsequent reuse, with a consistently low breakthrough of around 0.5% to 0.7% in all repeated rounds of beads reuse (Figure 4B). Furthermore, the harsher and more thorough regenerative wash did not negatively impact the performance of the magnetic affinity beads, as recoveries of about 70% were maintained in all elutions during repeated use of the beads. In fact, the harsher wash demonstrated better performance with higher overall recovery across multiple rounds of reuse compared to the gentler wash, especially in rounds 7 and 8, where the recovery of the gentler wash had dropped to less than 60% while the harsher wash’s recovery remained above 70%.

Next, we evaluated the effect of storage time on the magnetic affinity beads in the fridge to determine if degradation or reduced performance occurs with midterm to long term storage periods after regeneration using a harsher and more thorough regenerative wash. This may be helpful with labs wishing to recycle the beads, especially when purifying the same AAV over a period of time. We examined the recovery of AAV from the two scenarios that may be applicable: (1) magnetic affinity beads that were used once, regenerated, and stored at 4 °C over a period of 1, 3 and 6 months before subsequent use (Figure 4C), and (2) repeated use of the magnetic affinity beads that were regenerated before each round of reuse and stored at 4 °C over a period of 1, 3, and 6 months before the next use (Figure 4D). The data showed no reduction in magnetic affinity beads performance for purifying the AAV9 vector for at least 6 months.

### 2.4. Scalability of AAV9 Purification with AAV9 Magnetic Affinity Beads

With the promising results that we have obtained using viral supernatant produced from adherent packaging cells, we utilized clonal HEK293F-derived suspension cell cultures and animal-origin-free, chemically defined, serum-free media for the preparation of AAV9 viral supernatant and studied the translatability to larger-scale AAV production for potential pre-clinical applications. We upscaled the purification of AAV using magnetic affinity beads 40 times, from 0.5 mL to 20 mL of AAV9 viral supernatant, and compared the recoveries of parallel purifications.

**Figure 5:**
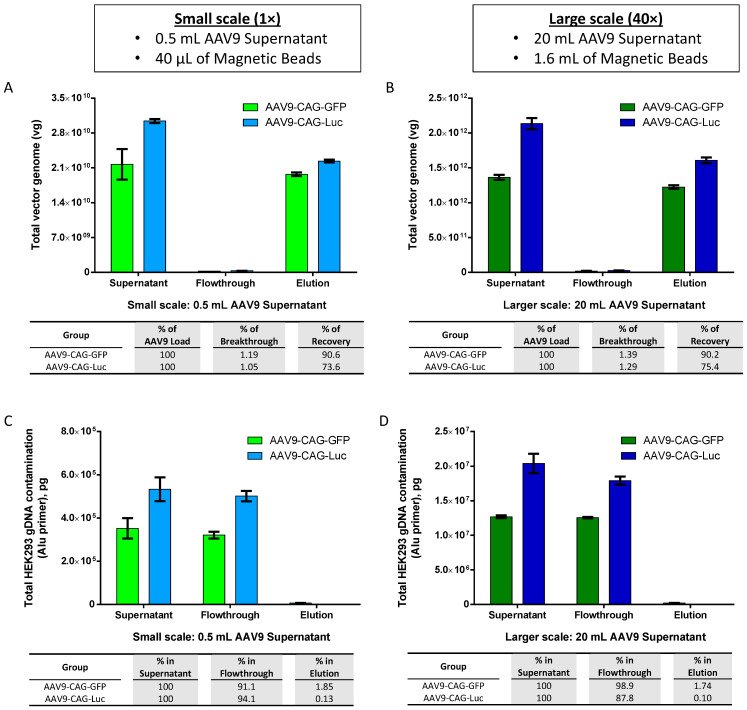

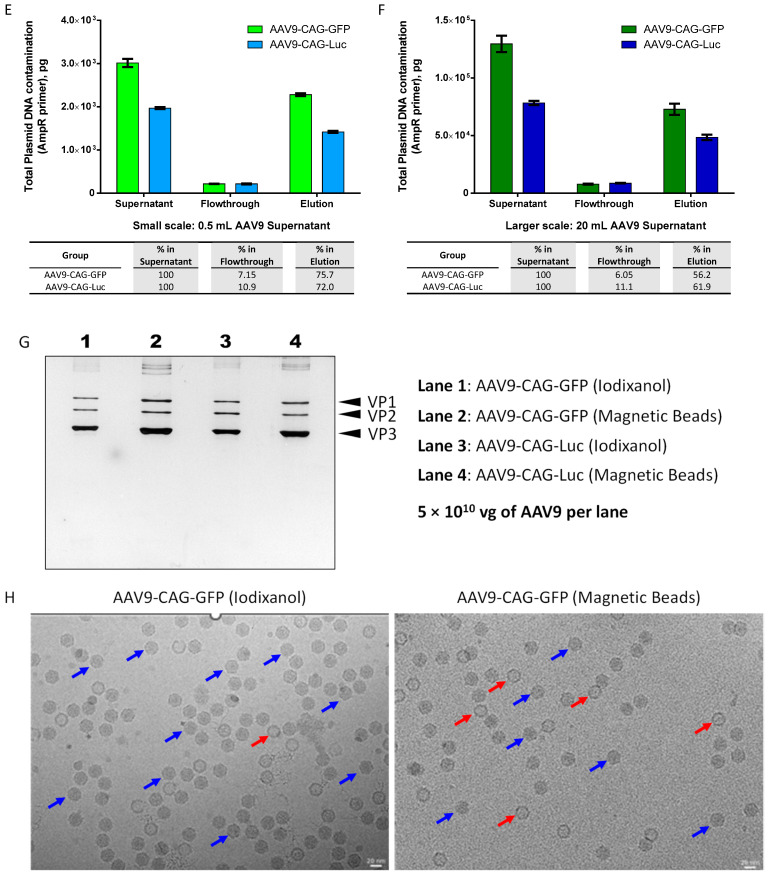
Scalability of magnetic affinity beads for AAV9 purification. (**A**,**B**) Upscaling of AAV9 purification with magnetic affinity beads (breakthrough and recovery percentages are indicated in the table below), (**C**,**D**) total HEK293 gDNA contamination, and (**E**,**F**) total plasmid DNA contamination (percentages of DNA contamination in flowthrough and elution are indicated in the table below) for small scale: 0.5 mL AAV9 supernatant (left panel: **A**,**C**,**E**) and larger scale: 20 mL AAV9 supernatant (right panel: **B**,**D**,**F**). Data are presented as mean (SD) (*n* = 3). (**G**) Silver-stained sodium dodecyl-sulfate polyacrylamide gel electrophoresis (SDS-PAGE) of AAV9 preparations purified by iodixanol ultracentrifugation (lanes 1 and 3) and by magnetic beads (lanes 2 and 4). (**H**) Typical cryo-electron micrographs (cryo-EM) of AAV9-CAG-GFP particles purified by iodixanol ultracentrifugation (left panel) and by magnetic beads (right panel). Scale bar: 20 nm. Blue arrows indicate AAV full capsids, and red arrows indicate AAV empty capsids.

The results depicted in Figure 5A,B indicated consistent recovery percentages of purified AAV9-CAG-GFP and AAV9-CAG-Luc between small and larger scales, with recovery rates ranging from 90.2% to 90.6% and 73.6% to 75.4%, respectively. Additionally, the corresponding total HEK293 gDNA contamination remained low in the elution, ranging from 0.1% to 1.85% for both small (Figure 5C) and larger scales (Figure 5D). However, quantification of total plasmid DNA contamination in the elution revealed higher levels of plasmid DNA contamination, ranging from 72% to 75.7% on a small-scale (Figure 5E) and 56.2% to 61.9% on a larger scale (Figure 5F).

The capsid purity of AAV9s purified with magnetic affinity beads was confirmed using silver-stained sodium dodecyl-sulfate polyacrylamide gel electrophoresis (SDS-PAGE) and compared to AAV9s purified by the iodixanol gradient centrifugation method (Figure 5G). Cryo-electron microscopy (cryo-EM) analysis was also performed on AAV9-CAG-GFP purified using magnetic affinity beads to distinguish between full and empty AAV capsids, and the results were compared to AAV9-CAG-GFP purified using the iodixanol gradient centrifugation method (Figure 5H). Unlike the iodixanol-gradient centrifugation method, which separates full AAV capsids from empty ones, magnetic affinity beads purify all AAV particles regardless of their capsid status. As shown in Table 1, AAV9-CAG-GFP purified using the iodixanol method consisted of 91.1% full AAV capsids and only 8.9% empty capsids, whereas AAV9-CAG-GFP purified using magnetic beads contained fewer full AAV capsids at 54.8% and more empty capsids at 45.2%.

An estimation of the theoretical percentage of AAV9-CAG-GFP full and empty capsids using UV A_260/280_ ratio measurement [19] revealed a comparable range of full versus empty AAV capsids as determined by cryo-EM analysis. For AAV9-CAG-Luc, only UV A_260/280_ ratio measurements were used to estimate full and empty AAV capsids (Table 1). Similar trends to those observed for AAV9-CAG-GFP were noted, albeit with slightly higher levels of full AAV capsids and slightly lower levels of empty AAV capsids in both iodixanol and magnetic beads-purified AAV9.

### 2.5. In Vivo Bioactivity of AAV9 Purification with AAV9 Magnetic Affinity Beads

To confirm the potency of magnetic affinity beads purified AAV9, we tail vein-injected 5 × 10^10^ vg/mouse of each magnetic affinity beads purified AAV9-CAG-GFP and AAV9-CAG-Luc into male C57BL/6JInv mice. Corresponding iodixanol-purified AAVs at the same dose were also included as positive controls.

**Figure 6:**
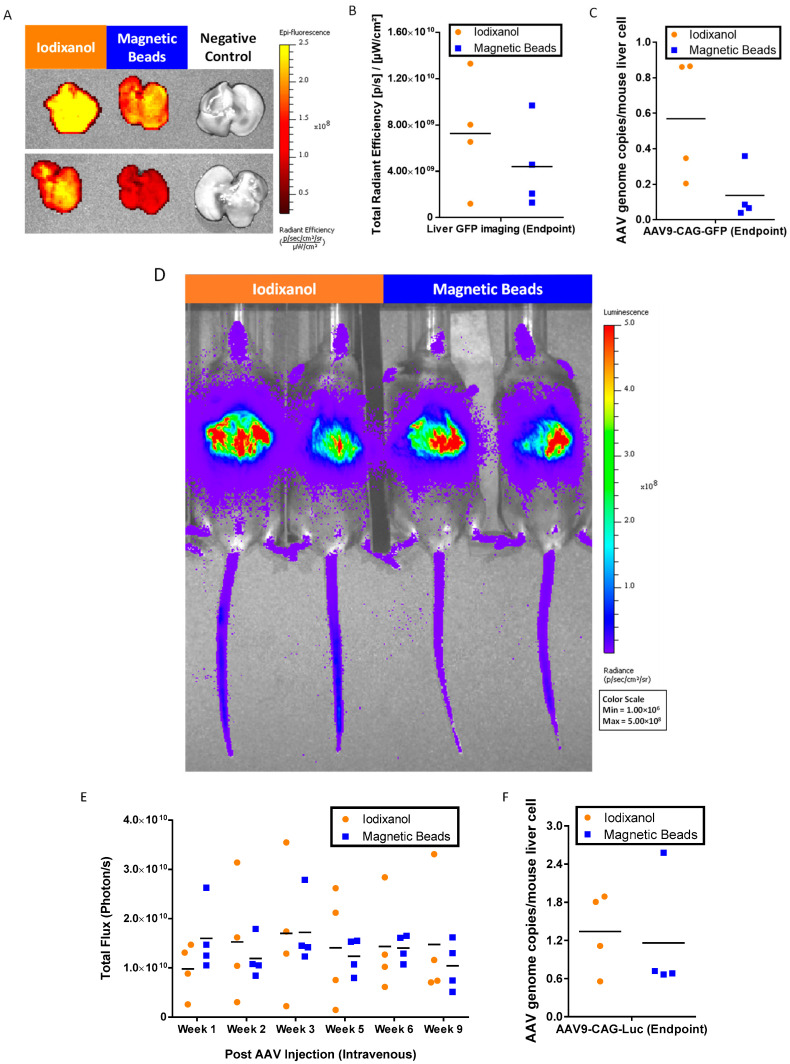
In vivo bioactivity of AAV9-purified by magnetic affinity beads compared to iodixanol-purified AAV9. Ex vivo liver green fluorescent protein (GFP) imaging: (**A**) representative images of liver GFP imaging acquired; (**B**) corresponding GFP total radiant efficiency; and (**C**) quantification of AAV9-CAG-GFP genome copies. Non-invasive luciferase imaging of whole mice: (**D**) representative images of luciferase imaging acquired; (**E**) corresponding luciferase total flux measurements from week 1 to week 9; and (**F**) quantification of AAV9-CAG-Luc genome copies at endpoint. Individual data points are presented, and mean values are indicated (*n* = 4).

At the endpoint, 9 weeks post injection of the AAVs, we harvested the livers and subjected them to ex vivo green fluorescent protein (GFP) imaging (Figure 6A) and GFP signal quantification (Figure 6B). The results indicated that AAV9-CAG-GFP purified using magnetic affinity beads was functional, and the quantitated GFP signals were approximately 1.65-fold less than iodixanol-purified AAV9-CAG-GFP. These findings were confirmed by liver AAV genome copy number quantification against the AAV9-CAG-GFP vector, which also showed lower gene copies in mice liver injected with magnetic affinity beads purified AAV9-CAG-GFP when compared to iodixanol purified AAV9-CAG-GFP (Figure 6C).

We monitored the luciferase signals in mice injected with AAVs using non-invasive imaging (Figure 6D). The luciferase signals from week 1 to week 9 were quantitated as shown in Figure 6E. Unlike the results of GFP imaging, luciferase signals between magnetic beads purified AAV9-CAG-Luc and iodixanol purified AAV9-CAG-Luc showed more similar intensity in vivo. This was further confirmed with liver AAV genome copy number quantification against the AAV9-CAG-Luc vector, which showed a similar mean value for magnetic affinity beads purified AAV9-CAG-Luc and iodixanol purified AAV9-CAG-Luc (Figure 6F).

Both GFP and luciferase imaging showed that magnetic beads purified from AAV9s were functional and expressed the desired transgenes in vivo. The purified AAV9s were comparable to iodixanol-purified AAV9s.

## 3. Discussion

The AAV viral vector has recently gained more popularity, not only on a large scale for clinical use [14] but also on a smaller scale for research purposes such as in vivo evaluation of potential therapeutic genes [20,21], generating in vivo disease models [22,23], ex vivo/in vitro gene delivery, and gene editing [24,25,26].

With the low recovery of AAV9 when using the AAVX affinity column [27] by others and us (Figure S1), we turned to evaluating the newly launched Dynabeads AAVX magnetic affinity beads (Thermo Fisher Scientific) to purify AAV9 on a smaller scale using the suggested protocol. Surprisingly, the AAVX magnetic affinity beads failed to purify AAV9 (Figure S2), suggesting that the AAVX affinity ligand has a very weak affinity for AAV9. The observation that the AAVX affinity column could still show some AAV9 binding and recovery was probably due to the target molecules (i.e., AAV9 viral particles) being brought into close contact with the beads in the chromatography column, while in the AAVX magnetic affinity beads purification, the target must ‘find the beads’ in suspension. This problem was resolved when the AAV9 magnetic affinity beads were used for purifying AAV9s.

In this study, AAV9 magnetic affinity beads have been shown to be effective in purifying total AAV9 particles with consistently high recovery between 70 and 90% (see Figure 1B, Figure 2A, Figure 4B and Figure 5A,B). However, when comparing the maximum binding capacity of AAV9 between purified AAV9 in PBS (Figure 1A; approximately 5 × 10^11^ total vg) and AAV9 viral supernatant (Figure 3C; approximately 1.2 × 10^11^ total vg), there was a 4.2-fold reduction in total AAV9 recovery when AAV9 crude viral supernatant was used. On the other hand, when evaluating the optimal binding capacity that resulted in minimal loss in flowthrough, purified AAV9 in PBS showed a value very close to its maximum capacity (i.e., 5 × 10^11^ total vg), at approximately 4.5 × 10^11^ total vg (Figure 1A). In contrast, for AAV9 viral supernatant, the optimal binding capacity was estimated to be only about 6.5 × 10^10^ total vg, almost 2-fold lower than its estimated maximum capacity at 1.2 × 10^11^ total vg (Figure 3C). This translated to an almost 7-fold reduction in total AAV9 recovery between AAV in PBS versus in crude viral supernatant. All results suggest that impurities in the sample load may affect the AAV9 binding on the AAV9 affinity ligand [28], requiring more magnetic beads to be utilized to ensure complete capture without leakage into the flowthrough.

Additionally, since AAV9 particles must ‘find the beads’ in suspension, we observed that a higher volume of AAV9 crude viral supernatant for every 40 μL magnetic slurry (approximately 1.2 mg magnetic beads) resulted in greater loss in the flowthrough (Figure 3A,B,D). Therefore, providing a sufficient amount of beads is essential to minimizing AAV9 loss in the flowthrough to less than or about 5%. Our data suggested that for every 40 μL of magnetic slurry (approximately 1.2 mg magnetic beads), the optimal sample volume should be within 0.5 to 1 mL of crude viral supernatant (refer to Figure 1B, Figure 2A, Figure 3A, Figure 4A and Figure 5A for 0.5 mL and Figure 3D for 1.0 mL). Such ratios may be important to ensure the success of upscaling purification from 0.5 mL to 20 mL of crude AAV9 viral supernatant using 1.6 mL magnetic affinity beads (Figure 5B).

In AAV purification steps using an affinity column with FPLC, genetically engineered endonucleases from Serratia marcescens, such as Benzonase (Merck, Darmstadt, Germany), Universal Nuclease (Pierce; Thermo Fisher Scientific), or UniversalBenzo Nuclease (Vazyme, Nanjing, China), are typically used to treat the AAV crude viral supernatant, removing DNA contamination before the purification process begins. However, this step significantly increases the cost of purification. Since AAV9 magnetic affinity beads purification steps are mainly performed in suspension, we compared the purity and yield in the presence or absence of endonuclease.

Our study indicated that when purifying AAV9 crude viral supernatant using AAV9 magnetic affinity beads, endonuclease treatment significantly reduced the leakage of AAV into the flowthrough, from 2.65% to 0.57% breakthrough (Figure 2A). This reduction is presumably due to the increased binding of AAV9 to its affinity ligands on the AAV9 magnetic beads after the removal of plasmid and gDNA, which otherwise could cause the AAV to aggregate [29] and potentially reduce the amount of AAV9 binding on its affinity ligand. Nevertheless, this leakage was negligible because the amount of loss was too small and did not significantly affect the total recovered AAV in elution (Figure 2A). This suggested that endonuclease treatment could be omitted, as it did not show an additional advantage but increased the purification cost.

Further analysis revealed that AAV9 magnetic affinity beads could efficiently remove 99.983% of host gDNA contamination from untreated crude viral supernatant, and endonuclease treatment only contributed to an additional 0.014% removal of host gDNA contamination, from 99.983% to 99.997% (Figure 2B). Efficient removal of gDNA without endonuclease treatment, up to 98.15% to 99.90%, was also observed when crude viral supernatant was prepared from suspension cell culture (Figure 5C,D).

It is worth noting that purified AAV9 contained plasmid DNA resistant to endonuclease digestion. As shown in Figure 2C, most of the endonuclease-resistant plasmid DNA (green bar) failed to be removed during the washing step (only 1.2% of plasmid DNA was found in the flowthrough), and the majority of the endonuclease-resistant plasmid DNA (77.4% in elution) was co-eluted with purified AAV9. Similar observations were found when crude viral supernatant from suspension cell culture without endonuclease treatment was used as the starting material (Figure 5E,F) as well as in iodixanol-purified AAV9s (Figure S3). The results also revealed that the crude viral supernatant contained very low levels of plasmid DNA contamination as compared to the lysate from suspension cell culture. Plasmid DNA may have been co-packaged with AAV9 particles, causing it to co-purify with AAV9 particles in the elution and not be found in the flowthrough. This phenomenon was also observed by Wright and colleagues when they were preparing the AAV2 viral vector, and they discussed that most of the nuclease-resistant nucleic acids were packaged into the AAV2 viral vector, with trace amounts possibly present on the AAV2 vector surface [29,30]. Further findings on the sources, implications, and strategies to reduce nucleic acid contamination from plasmid DNA used during AAV packaging as well as incomplete AAV vector genomes and host gDNA during AAV preparations for gene therapy have been reviewed elsewhere [31].

From our results, we propose that endonuclease treatment may not be necessary unless: (1) impurities in crude samples for purification have badly impacted the binding of AAV to its ligand, resulting in high flowthrough and low recovery; and (2) the gDNA contamination level is too high and not acceptable for use as the final product. In cases where further endonuclease treatment is needed, we recommend a further round of magnetic beads purification in the presence of endonuclease. In addition, the second round of purification could result in an increase in the capture and recovery of the partially purified AAV9, as demonstrated in Figure 1A.

In addition to the advantages of reducing costs by eliminating the need for endonuclease treatment on crude viral supernatant samples, AAV9 magnetic beads demonstrate high reusability and consistent performance, with recovery rates ranging from 69.3% to 78.6% and minimal loss in the flowthrough (0.5% to 0.7% breakthrough) over 8 rounds of reuse of the same beads, provided that the proper regeneration wash described in the Materials and Methods section was used. The regeneration wash used in this study was proven to be well-tolerated, as long-term storage at 4 °C and repeat regeneration showed no deterioration in the quality of magnetic affinity beads for reuse in purifying AAV9 (Figure 4C,D). Future evaluations of the reusability of magnetic affinity beads on a larger volume scale and with more repeat use rounds (beyond 100 rounds) [32] as well as cross-contamination [18] between each round will be interesting for a complete assessment of the beads’ reusability.

In this study, magnetic affinity beads purified from AAV9s were demonstrated to be functional (Figure 6). Reduced gene delivery and GFP signal in the liver were observed with magnetic beads purified AAV9-CAG-GFP compared to iodixanol purified AAV9-CAG-GFP (Figure 6A–C). This difference may be attributed to a higher number of empty capsids in the magnetic beads purified as AAV9-CAG-GFP, as indicated in Table 1. Several studies have shown that AAV empty capsids can affect in vivo gene delivery and increase toxicity [33,34,35]. However, there were no significant differences in the relative luminescence signals in vivo over time between magnetic beads purified AAV9-CAG-Luc and iodixanol purified AAV9-CAG-Luc (Figure 6D–F). This may be due to a lower number of AAV empty capsids in the magnetic beads purified AAV9-CAG-Luc preparation (Table 1). Therefore, a downstream removal of AAV empty capsids using methods such as iodixanol gradient centrifugation [36] or anion exchange chromatography with resins such as POROS HQ50 [37] and CIMmultus monolith [38] would enrich the recovery of full capsid AAV.

In summary, we have presented a new method for small-scale purification of AAV that is rapid, cost-effective, and does not require endonuclease treatment, while also allowing for repeat use (see Figure S4 for the new method overview of the entire AAV9 purification process using magnetic affinity beads). This affordable and efficient option will allow rapid screening for functional AAVs for different applications and targets, including in vivo administrations in rodents. Furthermore, our purification by magnetic beads could be adapted to industrial-scale single-step purification as described by Turco et al. [39], highlighting the potential cost-effective upscaling capability of AAVX and AAV9 magnetic affinity beads for future AAV purification and research endeavors.

## 4. Materials and Methods

### 4.1. Packaging of AAV9 Viral Vector

AAV9 vector particles (single-stranded AAV9) were produced using the 293T transient triple transfection method. This method involved the use of linear polyethylenimine (MW 25,000, Polysciences, Warrington, PA, USA), the HGT1 adenoviral helper plasmid, pAAV2/9n (a gift from James M. Wilson: Addgene plasmid # 112865; “http://n2t.net/addgene:112865 (accessed on 25 July 2024)”; RRID: Addgene_112865), and the AAV plasmid of either (1) pCAG/GFP (a gift from William Pu: Addgene plasmid # 139980; “http://n2t.net/addgene:139980 (accessed on 25 July 2024)”; RRID: Addgene_139980) to package AAV9-CAG-GFP or (2) pAAV-CAG-Luc (previously generated, unpublished data) to package AAV9-CAG-Luc. For the upscaling experiment from 0.5 mL of AAV9 viral supernatant to 20 mL of AAV9 viral supernatant, AAV9 vector particles were produced by the AAV-MAX Helper-Free AAV Production System Kit (Gibco; Thermo Fisher Scientific, Waltham, MA, USA), according to the manufacturer’s protocol.

### 4.2. Purification of the AAV9 Viral Vector Using the Iodixanol Density Gradient Centrifugation Method

On day 3 post-transfection, the cell pellet was harvested and lysed by subjecting it to five freeze-thaw cycles in TD buffer (PBS with 1 mM MgCl_2_ and 2.5 mM potassium chloride) containing 0.01% Pluronic F-68 (Gibco; Thermo Fisher Scientific, Waltham, MA, USA). The freeze-thaw process involved using liquid nitrogen and a 37 °C water bath. The lysate was then treated with 50 U/mL UniversalBenzo Nuclease (Vazyme, Nanjing, China) in the presence of 0.5% sodium deoxycholate (Sigma Aldrich, St. Louis, MO, USA) for 30 min. Afterward, the lysate was clarified by centrifugation at 10,000× *g* for 30 min and passed through a 0.45 µm polyethersulfone (PES) syringe filter (Merck, Darmstadt, Germany). Subsequently, the AAV9 viral particles were purified using the iodixanol density gradient centrifugation method previously described [40], employing OptiPrep iodixanol (PROGEN, Heidelberg, Germany). The vector particles were further purified to remove iodixanol contamination and then concentrated using an Amicon Ultra-4 100 K filter device (Merck, Darmstadt, Germany) with PBS (Gibco; Thermo Fisher Scientific, Waltham, MA, USA) containing 0.01% Pluronic F-68. The final volume of the purified vector particles was adjusted to 500 μL before being aliquoted in 50 μL portions per tube and stored at −80 °C.

### 4.3. Purification of AAV9 Using the Magnetic Affinity Beads Method

Transfected cells were replenished with fresh SF-DMEM (Sigma Aldrich, St. Louis, MO, USA) after 24 h post-transfection. On day 6 post-transfection, crude AAV9 viral supernatant was harvested and filtered through a 0.2 µm PES filter unit (Nalgene; Thermo Fisher Scientific, Waltham, MA, USA).

For small-scale purification, 0.5 mL of filtered crude viral supernatant (i.e., supernatant containing 0.01% Pluronic F-68) was added to 40 µL of Dynabeads CaptureSelect AAV9 magnetic affinity beads (Thermo Fisher Scientific, Waltham, MA, USA) and incubated at room temperature while mixing using a Labquake tube rotator (Barnstead Thermolyne, Dubuque, IA, USA) for 30 min. After incubation, the beads were collected with a DynaMag-2 magnet magnetic stand (Invitrogen; Thermo Fisher Scientific, Waltham, MA, USA), and the supernatant (i.e., flowthrough) was removed. The beads were then washed twice with 500 μL of wash buffer (PBS with 150 mM NaCl) before eluting twice with 40 μL of elution buffer (50 mM citric acid, pH 2.5 with 0.01% Pluronic F-68) and incubated for 10 min at room temperature with occasional mixing. Each 40 μL of eluted AAV9 (i.e., elution) was immediately neutralized with 4.4 μL of neutralization buffer (2M Tris, pH 9.0).

For preparing sufficient AAV9 for animal work, magnetic affinity beads purification was upscaled (40-fold) and performed with about 20 mL of filtered crude viral supernatant containing 0.01% Pluronic F-68 and 1.6 mL of Dynabeads CaptureSelect AAV9 magnetic affinity beads. After 30 min of binding with the tube rotator at room temperature, the beads were collected with a DynaMag-50 magnet magnetic stand (Invitrogen; Thermo Fisher Scientific, Waltham, MA, USA) and washed twice with 20 mL of wash buffer before being eluted twice with 1.6 mL of elution buffer at conditions such as small-scale purification. The eluted AAV9 was immediately neutralized with 176 μL of neutralization buffer and further concentrated and buffer exchanged to PBS containing 0.01% Pluronic F-68 using the Amicon Ultra-4 100 K filter device to a final volume of 500 μL before being aliquoted into 50 μL per tube and stored at −80 °C.

### 4.4. Regeneration Wash of Dynabeads CaptureSelect AAV9 Magnetic Affinity Beads

Immediately after the elution step, AAV9 magnetic affinity beads were subjected to a regeneration wash using 0.5 mL of 6M Guanidine HCl (Sigma Aldrich, St. Louis, MO, USA) for every 40 µL of magnetic affinity beads. The tube was mixed continuously using a vortex mixer (Heathrow Scientific, Vernon Hills, IL, USA) and shaken at minimum speed for 10 min. This was followed by another 2 rounds of washing with 0.5 mL of wash buffer (PBS with 150 mM NaCl) at the same setting for 1 min per round before the magnetic beads were ready for the next purification.

### 4.5. Titration of the AAV9 Viral Vector by Quantitative PCR

Quantitative PCR was conducted using an *Xho*I-linearized pCAG/GFP plasmid as a reference standard. This reference standard was subjected to a 10-fold serial dilution, ranging from 1 × 10^1^ to 1 × 10^8^ copies/μL (in 5 μL samples), in a 20 μL PCR reaction. All DNAs were diluted in IDTE, pH 8.0 (10 mM Tris, 0.1 mM EDTA; Integrated DNA Technologies, Coralville, IA, USA), containing 0.01% Pluronic F68. The PCR was performed with EvaGreen qPCR master mix (Biotium, Fremont, CA, USA) and qPCR primers (synthesized by Integrated DNA Technologies, Coralville, IA, USA) against the CAG promoter (i.e., forward primer, CAG F1: 5′-GTCAATGGGTGGAGTATTTACGG-3′, and reverse primer, CAG R1: 5′-AGGTCATGTACTGGGCATAATGC-3′). The thermal cycling conditions consisted of one 2-min cycle at 95 °C followed by forty 2-step cycles (5 s at 95 °C and 20 s at 60 °C). The PCR was conducted using a Rotor-Gene Q real-time PCR cycler (QIAGEN, Valencia, CA, USA) and analyzed with its bundled Rotor-Gene Q software version: 2.0.2 (Build 4).

When titering samples with plasmid DNA contamination (such as viral supernatant and flowthrough), nuclease treatment was performed before qPCR using 90 U/mL Universal Nuclease (Pierce; Thermo Fisher Scientific, Waltham, MA, USA) in the presence of 2 mM MgCl_2_ in PBS for 30 min in a 37 °C water bath.

### 4.6. Estimation of Total HEK293 gDNA Contamination by Quantitative PCR

Quantitative PCR was conducted using HEK293 DNA control (Lot 2209026; Applied Biosystems; Thermo Fisher Scientific, Waltham, MA, USA) as the reference standard. A 10-fold serial dilution of the reference standard, ranging from 0.003 to 300 pg/μL (in 10 μL samples), was used in a 30 μL PCR reaction. The PCR was performed with qPCR primers against the *Alu* sequence [41] (i.e., forward primer, Alu F101: 5′-GGTGAAACCCCGTCTCTACT-3′ and reverse primer, Alu R206: 5′-GGTTCAAGCGATTCTCCTGC-3′) under similar conditions as described in the Section 4.5 method.

### 4.7. Estimation of Total Plasmid DNA Contamination by Quantitative PCR

Quantitative PCR was conducted using the *Hind*III-linearized pAAV-HLP-hINSco plasmid [42] as the reference standard. A 10-fold serial dilution of the reference standard, ranging from 3 to 3 × 10^7^ copies/μL (in 10 μL samples), was used in a 30 μL PCR reaction. The PCR was performed with qPCR primers against the ampicillin resistance (*AmpR*) gene sequence (i.e., forward primer, AmpR F619: 5′-GACTGGATGGAGGCGGATA-3′ and reverse primer, AmpR R768: 5′-GATACGGGAGGGCTTACCA-3′), under similar conditions as described in the Section 4.5 method.

### 4.8. SDS-PAGE and Silver Stain

AAV9 vector purity (5 × 10^1^⁰ vg per AAV9 per lane in a 20 µL loading sample) was assessed using 10% SDS-PAGE (Bio-Rad, Hercules, CA, USA) and silver staining (Pierce; Thermo Fisher Scientific, Waltham, MA, USA) according to the manufacturers’ instructions. Silver-stained SDS-PAGE gel was imaged using iBright FL1500 imaging system (Invitrogen; Thermo Fisher Scientific, Waltham, MA, USA).

### 4.9. Cryo-Electron Microscopy Grid Preparation, Data Collection, and Image Processing

Three µL of concentrated AAV9 was applied to glow-discharged cryo-EM grids (CF-2/1-3CU, Protochips, Morrisville, NC, USA). The grids were plunge-frozen in liquid ethane using a Vitrobot (Thermo Fisher Scientific, Waltham, MA, USA) or Leica EM GP2 (Leica Microsystems, Wetzlar, Germany) and stored in liquid nitrogen for subsequent imaging. Cryo-EM grids were loaded into a Titan Krios transmission electron microscope (TEM) (Thermo Fisher Scientific, Waltham, MA, USA) operated at 300 kV and equipped with a BioQuantum K3 direct electron detector (Gatan, Pleasanton, CA, USA). Movies of AAV9 particles were automatically recorded using SerialEM [43] and processed in RELION-4 [44]. Imported movies were motion-corrected and dose-weighted using RELION’s implementation of the MotionCor2 algorithm [45]. The contrast transfer function (CTF) was estimated by CTFFIND-4 [46], and particles were first automatically picked using Gautomatch “https://github.com/JackZhang-Lab/Gautmatch (accessed on 25 July 2024)”. Extracted particles were subjected to 2D classification to remove any contaminating features. Particles were then automatically picked by reference-based template-matching in RELION-4, using a selected 2D class as a 2D reference. Extracted particles were subjected to further 2D classification. Subsequently, two rounds of 3D classification without symmetry were performed to assess the ratio of AAV9 particles with full and empty capsids.

### 4.10. UV A_260/280_ Ratio Determination

Purified AAV (1.5 µL) was measured undiluted in a NanoDrop ND-1000 (Thermo Fisher Scientific, Waltham, MA, USA) at UV wavelengths of 260 nm and 280 nm. PBS, used to resuspend the AAV, was used to zero the instrument before each sample measurement. The A_260_ was divided by the A_280_ to calculate the UV A_260_/_280_ ratio.

### 4.11. In Vivo Experiments

All animal experiments were conducted in accordance with the guidelines and protocols approved by the Institutional Animal Care and Use Committee (IACUC) of the National University of Singapore. The mice were housed in a specific pathogen-free facility within the university, where they were subjected to regular 12-h light/dark cycles and provided with ad libitum access to standard feed and water. Male C57BL/6JInv (InVivos, Singapore) mice, aged 8 to 12 weeks, were used for the AAV9 injection. Each mouse received a total of 1 × 10^11^ vg of AAV9 consisting of 5 × 10^10^ vg/mouse of AAV9-CAG-GFP and 5 × 10^10^ vg/mouse of AAV9-CAG-Luc, which were either iodixanol-purified AAV9s or magnetic affinity beads-purified AAV9s, administered via the tail vein. Three mice were assigned to each group, and each mouse was given a specific code number for tracking and record-keeping. At the experimental endpoint, mice were humanely euthanized by inducing hypoxia using carbon dioxide, followed by cervical dislocation, in accordance with IACUC guidelines. Liver tissues were promptly harvested in RNAlater (Invitrogen; Thermo Fisher Scientific, Waltham, MA, USA) for organ preservation and stored at −80 °C. All mice in this study remained healthy and survived until the endpoint, and every effort was made to minimize any potential suffering.

### 4.12. In Vivo Non-Invasive Luciferase Imaging

Mice were intraperitoneally injected with 160 mg/kg D-luciferin (PerkinElmer, Waltham, MA, USA) dissolved in saline before luciferase imaging using the IVIS Spectrum In Vivo Imaging System (PerkinElmer, Waltham, MA, USA). The mice were anesthetized with 1–2.5% isoflurane, supplied with 2 L/min oxygen, and 9 images were acquired at medium binning, F/stop of 4, and 1-s exposure time with a 1-min interval wait time for each imaging image. The data were analyzed using Living Image version 4.7.3 (PerkinElmer, Waltham, MA, USA). The region of interest (ROI) covering the liver area was defined, and quantification of light emission in total flux was performed. The peak signal for each mouse was used as the data point for the experiment. 

### 4.13. Ex Vivo Liver GFP Imaging

Mice livers were harvested and immediately placed in the IVIS Spectrum In Vivo Imaging System (PerkinElmer, Waltham, MA, USA) for GFP imaging. Images were acquired using various combinations of excitation lights (i.e., 465 and 500 nm) and emission filters (i.e., 520, 540, 560, and 500 nm) at medium binning, F/stop of 2, and automatic exposure time. The data were analyzed using Living Image version 4.7.3 (PerkinElmer, Waltham, MA, USA). Control uninjected liver samples were included to assist in the spectral unmixing procedure for removing the autofluorescence background from the GFP signal. Total radiant efficiency for each liver was finally quantified using the unmixed images with ROI tools.

### 4.14. AAV Viral Genome Copies Quantification in the Liver

Total gDNA from the livers of treated mice was automatically extracted by TGuide S16 (TIANGEN, Beijing, China) equipped with the reagent TGuide Smart Magnetic Tissue DNA Kit (TIANGEN, Beijing, China) according to the manufacturer’s protocol. AAV viral genome copy number was determined by real-time qPCR using EvaGreen qPCR master mix (Biotium, Fremont, CA, USA) with a Rotor-Gene Q real-time PCR cycler (QIAGEN, Valencia, CA, USA) and analyzed with its bundled Rotor-Gene Q software version: 2.0.2 (Build 4). For AAV9-CAG-GFP viral genome copy quantification, the PCR primers were designed against the *eGFP* gene (i.e., forward primer, GFP F363: 5′-GAACCGCATCGAGCTGAA-3′, and reverse primer, GFP R473: 5′-TGCTTGTCGGCCATGATATAG-3′). Whereas, for AAV9-CAG-Luc viral genome copy quantification, the PCR primers were designed against the *luciferase* gene (i.e., forward primer, qLuc_F: 5′-CCAGGGATTTCAGTCGATGT-3′, and reverse primer, qLuc_R: 5′-AATCTCACGCAGGCAGTTCT-3′). The results were then normalized with the mouse glyceraldehyde-3-phosphate dehydrogenase (*mGAPDH*) housekeeping gene (i.e., forward primer, mGAPDH F: 5′-TGGAGAGCCCGCTCAGACCC-3′, and reverse primer, mGAPDH R: 5′-GGATTGGGTGTCCCTGCGGCC-3′) to calculate AAV genome copies/mouse liver cell.

### 4.15. Statistical Analyses

The number of replicates or animals used in each group is provided in the respective figure legends. All values are presented as the mean (SD). Statistical significance in studies involving two groups was determined using the Student’s unpaired *t*-test, where ‘ns’ indicates not significant, * indicates *p* < 0.05 and is considered statistically significant, and **** indicates *p* < 0.0001 and is considered extremely significant.

## Figures and Tables

**Table 1:** Cryo-electron microscopy (cryo-EM) and UV A_260_/_280_ ratio analysis of AAV9 particles. Cryo-EM data were derived from the 3D classification of 46,095 AAV9-CAG-GFP particles purified by iodixanol ultracentrifugation and 7814 AAV9-CAG-GFP particles purified by magnetic beads. The UV A_260_/_280_ ratio of AAV9 samples was indicated, and the theoretical % of AAV full capsids and AAV empty capsids was estimated. N.D. indicates “not determined”.

Group	Cryo-EM (Full)	Cryo-EM (Empty)	UV A_260/280_ (Full)	UV A_260/280_ (Empty)
**AAV9-CAG-GFP (Iodixanol)**	91.1%	8.9%	80–91% (A_260/280_ ratio = 1.348)	9–20% (A_260/280_ ratio = 1.348)
**AAV9-CAG-GFP (Magnetic Beads)**	54.8%	45.2%	50–67% (A_260/280_ ratio = 1.164)	33–50% (A_260/280_ ratio = 1.164)
**AAV9-CAG-Luc (Iodixanol)**	N.D.	N.D.	91–100% (A_260/280_ ratio = 1.381)	0-9% (A_260/280_ ratio = 1.381)
**AAV9-CAG-Luc (Magnetic Beads)**	N.D.	N.D.	67–80% (A_260/280_ ratio = 1.287)	20–33% (A_260/280_ ratio = 1.287)

## Data Availability

The raw data supporting the conclusions of this article will be made available by the authors on request.

## Supplementary Materials

The following supporting information can be downloaded at: https://www.mdpi.com/article/10.3390/ijms25158342/s1.
