# Supplementary material for: Efficient AAV9 Purification Using a Single-Step AAV9 Magnetic Affinity Beads Isolation
Source: Int J Mol Sci. 2024 Jul 30;25(15):8342. doi: 10.3390/ijms25158342 (PMC11313462; doi:10.3390/ijms25158342)
Supplement: Supplementary file 1 [file ijms-25-08342-s001.zip › ijms-3104777-supplementary.pdf]

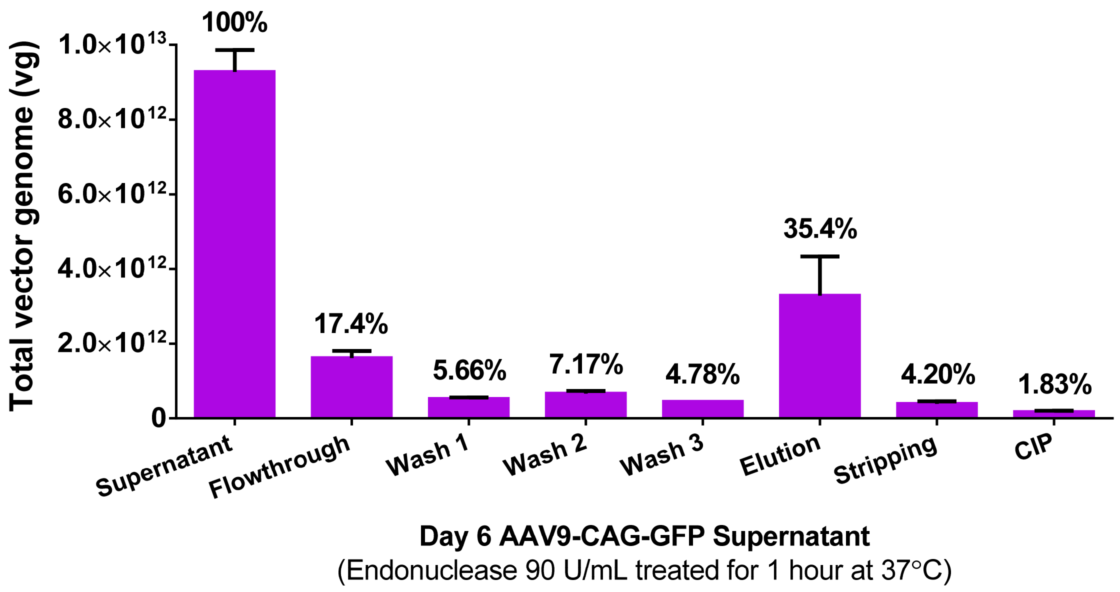

Figure S1. Purification of AAV9-CAG-GFP from day 6 endonuclease-treated crude viral supernatant in serum-free DMEM (~200 mL) using a POROS AAVX affinity column with an AKTA pure 25M chromatography system. Data are presented as mean (SD) (n = 2).

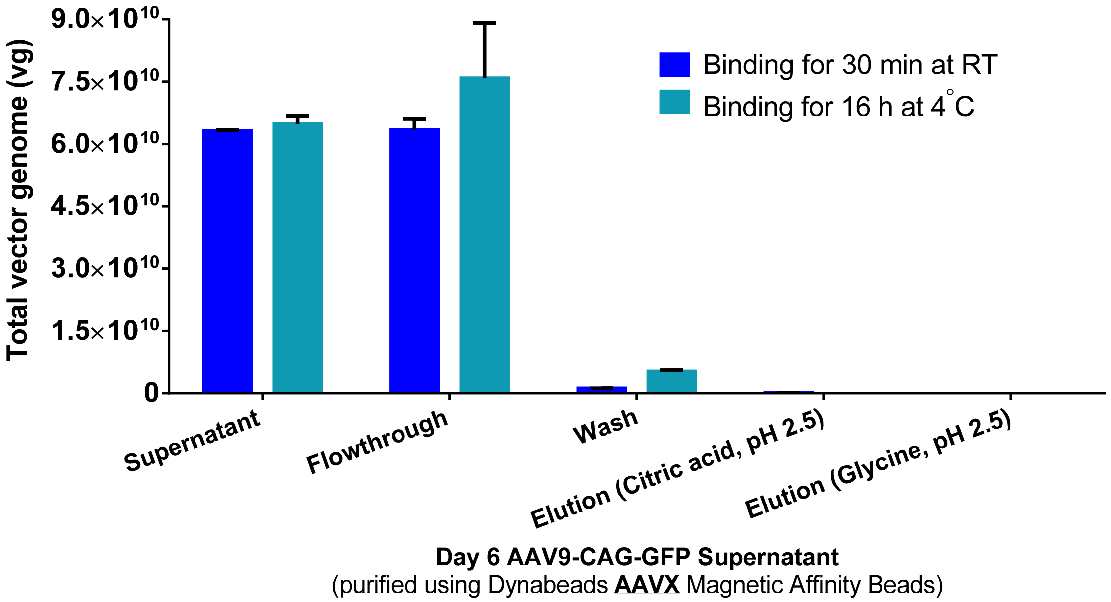

Group	% of AAV9 Load	% of Breakthrough	% of Wash	% of Recovery (Citric acid)	% of Recovery (Glycine)
30 min at RT	100	100.6	1.84	0.167	0.017
16 h at 4°C	100	116.9	8.13	0.001	0.002

Figure S2. Purification of day 6 AAV9-CAG-GFP viral supernatant in serum-free DMEM using Dynabeads AAVX magnetic affinity beads. Each purification used 0.5 mL AAV9 viral solution and 40 μL AAVX magnetic beads. The standard binding time between AAV9 and magnetic beads, 30 minutes at RT, was increased to 16 hours at 4° C for comparison, confirming no binding of AAV9 to AAVX magnetic beads. Breakthrough, loss in wash, and recovery percentages are indicated in the table below. Data are presented as mean (SD) (n = 2).

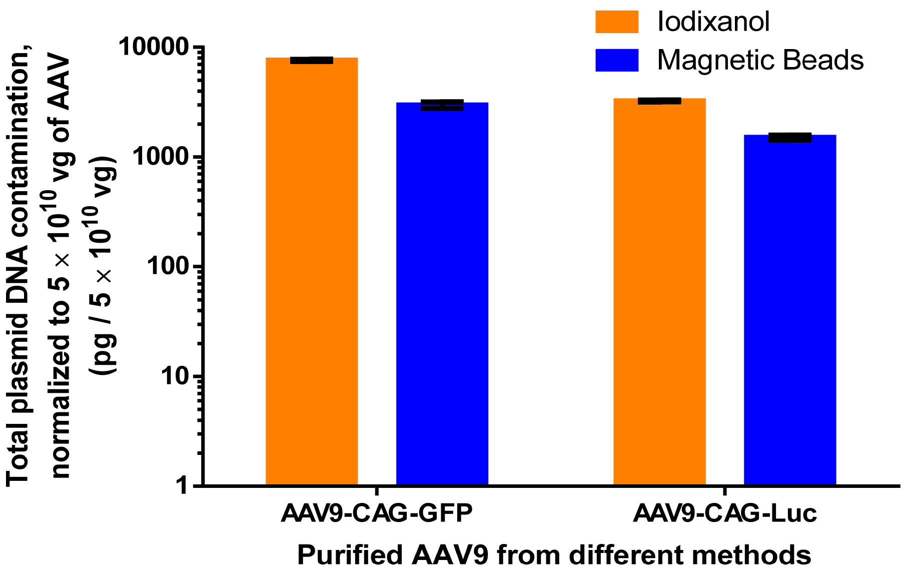

Figure S3. Total plasmid DNA contamination in purified AAV9-CAG-GFP and AAV9-CAG-Luc using either iodixanol centrifugation method or magnetic beads method. Data are presented as mean (SD) (n = 3).

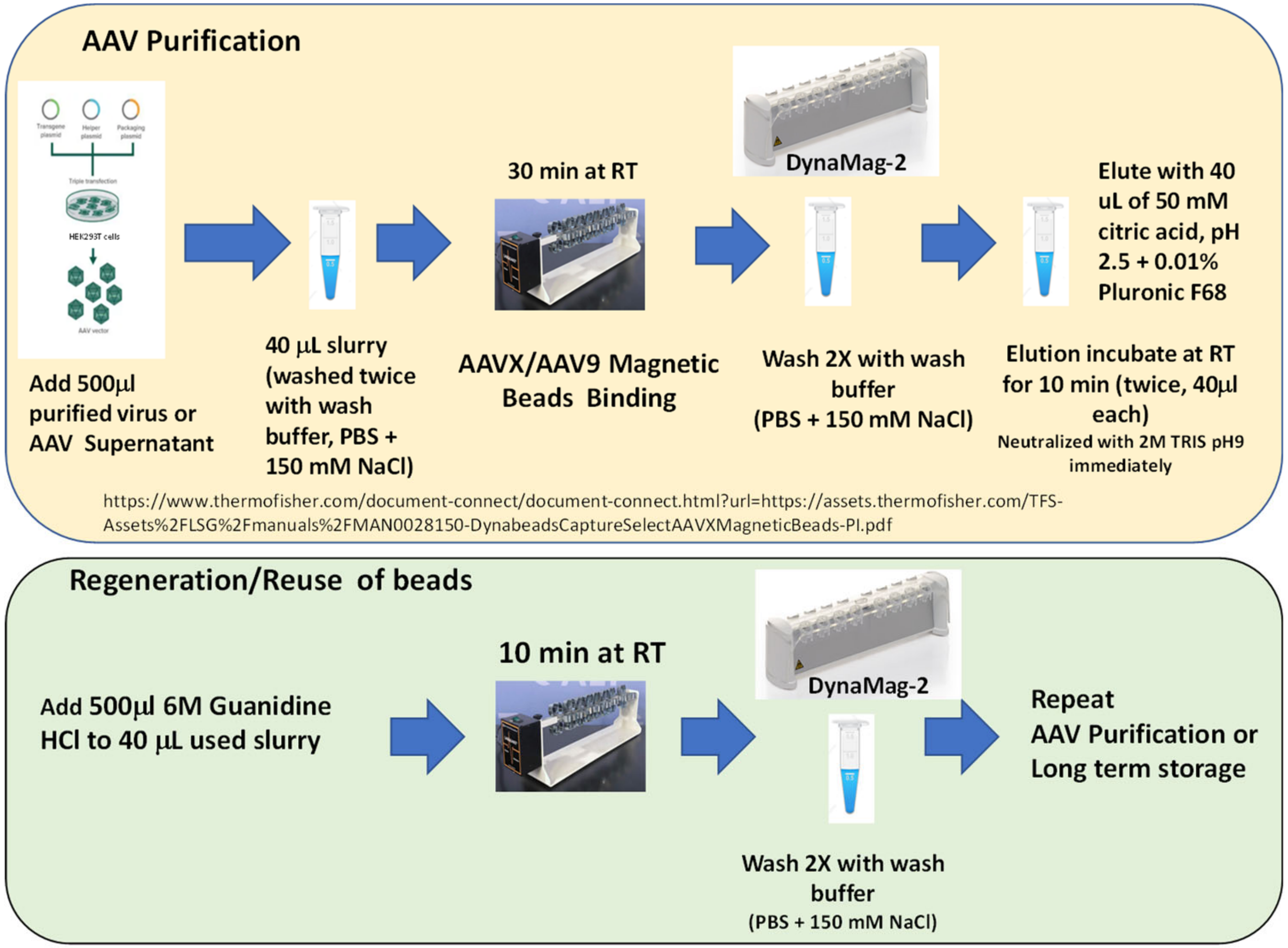

Figure S4. Schematic diagram provides an overview of the entire AAV9 purification process using magnetic affinity beads, including the regeneration and reuse of the beads. The protocol in the yellow box outlines the method used in this study, which is identical to the Thermo Fisher Scientific method. The protocol in the green box details the optimized method developed in this study for the regeneration and reuse of the beads.

Table S1 Clinical Trials involving AAV9 Gene Therapy.

Conditions	NCT Number	Study Title	Study URL
CLN7	NCT04737460	Study for the Treatment for CLN7 Disease	https://clinicaltrials.gov/study/NCT04737460
Neuronal Ceroid Lipofuscinosis Type 2	NCT05791864	A First-in-Human Study in Pediatric Patients With Ocular CLN2 Disease	https://clinicaltrials.gov/study/NCT05791864
CLN3|Batten Disease	NCT03770572	Gene Therapy for Children With CLN3 Batten Disease	https://clinicaltrials.gov/study/NCT03770572
Variant Late-Infantile Neuronal Ceroid Lipofuscinosis	NCT02725580	Gene Therapy For Children With Variant Late Infantile Neuronal Ceroid Lipofuscinosis 6 (vLINCL6) Disease	https://clinicaltrials.gov/study/NCT02725580
Canavan Disease	NCT04998396	A Study of AAV9 Gene Therapy in Participants With Canavan Disease	https://clinicaltrials.gov/study/NCT04998396
Canavan Disease	NCT04126005	Natural History Study of Patients With Canavan Disease	https://clinicaltrials.gov/study/NCT04126005
Infantile-onset Pompe Disease	NCT05567627	Clinical Exploration of Adeno-associated Virus (AAV) Expressing Human Acid Alpha- Glucosidase (GAA) Gene Therapy for Patients With Infantile-onset Pompe Disease	https://clinicaltrials.gov/study/NCT05567627
Pompe Disease Infantile-Onset	NCT05793307	Evaluation of the Safety and Efficacy of Infantile-onset Pompe Disease Gene Therapy Drug	https://clinicaltrials.gov/study/NCT05793307
Pompe Disease (Late-onset)	NCT06391736	Evaluation of the Safety and Efficacy of Late-onset Pompe Disease Gene Therapy Drug	https://clinicaltrials.gov/study/NCT06391736
NGLY1 Deficiency	NCT06199531	Safety and Efficacy of GS-100 Gene Therapy in Patients With NGLY1 Deficiency	https://clinicaltrials.gov/study/NCT06199531
Spinal Muscular Atrophy Type I	NCT05614531	Clinical Trial to Assess the Safety and Efficacy of EXG001-307 in Patients With Spinal Muscular Atrophy Type 1	https://clinicaltrials.gov/study/NCT05614531
Spinal Muscular Atrophy	NCT03381729	Study of Intrathecal Administration of Onasemnogene Abeparvovec-xioi for Spinal Muscular Atrophy	https://clinicaltrials.gov/study/NCT03381729
Spinal Muscular Atrophy 1	NCT02122952	Gene Transfer Clinical Trial for Spinal Muscular Atrophy Type 1	https://clinicaltrials.gov/study/NCT02122952
Spinal Muscular Atrophy	NCT03955679	AveXis Managed Access Program Cohort for Access to AVXS-101	https://clinicaltrials.gov/study/NCT03955679
SMA	NCT03461289	Single-Dose Gene Replacement Therapy Clinical Trial for Participants With Spinal Muscular Atrophy Type 1	https://clinicaltrials.gov/study/NCT03461289
Spinal Muscular Atrophy	NCT05824169	Evaluation of Safety and Efficacy of Gene Therapy Drug in the Treatment of Spinal Muscular Atrophy (SMA) Type 1 Patients	https://clinicaltrials.gov/study/NCT05824169
Spinal Muscular Atrophy Type I	NCT03837184 (continuation of study to NCT02122952)	Single-Dose Gene Replacement Therapy Using for Patients With Spinal Muscular Atrophy Type 1 With One or Two SMN2 Copies	https://clinicaltrials.gov/study/NCT03837184
Spinal Muscular Atrophy	NCT03505099	Pre-Symptomatic Study of Intravenous Onasemnogene Abeparvovec-xioi in Spinal Muscular Atrophy (SMA) for Patients With Multiple Copies of SMN2	https://clinicaltrials.gov/study/NCT03505099
SMA II	NCT05901987	Evaluation of Safety and Efficacy of Gene Therapy Drug in the Treatment of Spinal Muscular Atrophy (SMA) Type 2 Patients	https://clinicaltrials.gov/study/NCT05901987
SMA - Spinal Muscular Atrophy|Gene Therapy	NCT03306277 (continuation of study to NCT02122952 & NCT03837184)	Gene Replacement Therapy Clinical Trial for Participants With Spinal Muscular Atrophy Type 1	https://clinicaltrials.gov/study/NCT03306277
Duchenne Muscular Dystrophy	NCT04240314	AAV9 U7snRNA Gene Therapy to Treat Boys With DMD Exon 2 Duplications.	https://clinicaltrials.gov/study/NCT04240314
Duchenne Muscular Dystrophy	NCT03362502	A Study to Evaluate the Safety and Tolerability of PF-06939926 Gene Therapy in Duchenne Muscular Dystrophy	https://clinicaltrials.gov/study/NCT03362502
Oculopharyngeal Muscular Dystrophy	NCT06185673	A Study to Evaluate the Safety and Clinical Activity of Intramuscular Doses of BB-301 Administered to Subjects With Oculopharyngeal Muscular Dystrophy With Dysphagia	https://clinicaltrials.gov/study/NCT06185673
Danon Disease	NCT06092034	A Multi-Center, Open Label Gene Therapy Study of RP-A501 in Male Patients With Danon Disease	https://clinicaltrials.gov/study/NCT06092034
Arrhythmogenic Right Ventricular Cardiomyopathy	NCT06228924	Open-label, Dose Escalation Study of Safety and Preliminary Efficacy of TN-401 in Adults With PKP2 Mutation-associated ARVC	https://clinicaltrials.gov/study/NCT06228924
Danon Disease	NCT03882437	Gene Therapy for Male Patients With Danon Disease (DD) Using RP-A501; AAV9.LAMP2B	https://clinicaltrials.gov/study/NCT03882437
Heart Failure|Dilated Cardiomyopathy	NCT05837143	Myocardial Telomere Recapping Study for Dilated Cardiomyopathy	https://clinicaltrials.gov/study/NCT05837143
Arrhythmogenic Right Ventricular Cardiomyopathy	NCT06311708	Non-interventional Study of Seroprevalence of Pre-existing Antibodies Against Adenovirus-associated Virus Vector (AAV9) and the Progression of Disease in Patients With Plakophilin 2 (PKP2)-Associated Arrhythmogenic Right Ventricular Cardiomyopathy (ARVC)	https://clinicaltrials.gov/study/NCT06311708
Hypertrophic Cardiomyopathy	NCT05836259	Study of Safety and Tolerability of TN-201 in Adults With Symptomatic MYBPC3 Mutation-associated HCM	https://clinicaltrials.gov/study/NCT05836259
Household Contacts	NCT04543357	A Study to Evaluate AAV9 Neutralizing Antibody Seroconversion in Household Contacts.	https://clinicaltrials.gov/study/NCT04543357
Mesial Temporal Lobe Epilepsy	NCT06063850	AMT-260 Gene Therapy Study in Adults With Unilateral Refractory Mesial Temporal Lobe Epilepsy	https://clinicaltrials.gov/study/NCT06063850
Rett Syndrome	NCT06152237	Safety and Efficacy of TSHA-102 in Pediatric Females With Rett Syndrome (REVEAL Pediatric Study)	https://clinicaltrials.gov/study/NCT06152237
Rett Syndrome	NCT05606614	Safety and Efficacy of TSHA-102 in Adult Females With Rett Syndrome (REVEAL Adult Study)	https://clinicaltrials.gov/study/NCT05606614
HIV-1-infection	NCT05144386	Study of EBT-101 in Aviremic HIV-1 Infected Adults on Stable ART	https://clinicaltrials.gov/study/NCT05144386
Type II Gaucher Disease	NCT06272149	An Exploratory Clinical Trial of VGN-R08b in Patients With Type II Gaucher Disease	https://clinicaltrials.gov/study/NCT06272149
SMARD1|CMT2S	NCT05152823	Gene Therapy for IGHMBP2-Related Diseases	https://clinicaltrials.gov/study/NCT05152823
Frontotemporal Dementia|FTD|FTD-GRN|Dementia, Frontotemporal	NCT06064890	A Study to Evaluate the Safety and Effect of AVB-101, a Gene Therapy Product, in Subjects With a Genetic Sub-type of Frontotemporal Dementia (FTD-GRN)	https://clinicaltrials.gov/study/NCT06064890
Dravet Syndrome	NCT06283212	A Clinical Study to Evaluate the Safety and Efficacy of ETX101, an AAV9-Delivered Gene Therapy in Children With SCN1A-positive Dravet Syndrome	https://clinicaltrials.gov/study/NCT06283212
Rett Syndrome	NCT05898620	A Novel, Regulated Gene Therapy (NGN-401) Study for Female Children With Rett Syndrome	https://clinicaltrials.gov/study/NCT05898620
Dravet Syndrome	NCT06112275	A Clinical Study to Evaluate the Safety and Efficacy of ETX101, an AAV9-Delivered Gene Therapy in Children With SCN1A-positive Dravet Syndrome (Australia Only)	https://clinicaltrials.gov/study/NCT06112275
Infantile GM2 Gangliosidosis (Disorder)	NCT04798235	First-in-Human Study of TSHA-101 Gene Therapy for Treatment of Infantile Onset GM2 Gangliosidosis	https://clinicaltrials.gov/study/NCT04798235
Lysosomal Diseases|Gangliosidosis|GM1	NCT03952637	A Phase 1/2 Study of Intravenous Gene Transfer With an AAV9 Vector Expressing Human Beta-galactosidase in Type I and Type II GM1 Gangliosidosis	https://clinicaltrials.gov/study/NCT03952637
